# On the permittivity of titanium dioxide

**DOI:** 10.1038/s41598-021-92021-5

**Published:** 2021-06-14

**Authors:** Julie Bonkerud, Christian Zimmermann, Philip Michael Weiser, Lasse Vines, Eduard V. Monakhov

**Affiliations:** Physics Department, Centre for Materials Science and Nanotechnology, University of Oslo, Blindern, P.O. Box 1048, 0316 Oslo, Norway

**Keywords:** Electronic properties and materials, Materials for devices, Materials for energy and catalysis

## Abstract

Conductive rutile TiO_2_ has received considerable attention recently due to multiple applications. However, the permittivity in conductive, reduced or doped TiO_2_ appears to cause controversy with reported values in the range 100–10,000. In this work, we propose a method for measurements of the permittivity in conductive, n-type TiO_2_ that involves: (i) hydrogen ion-implantation to form a donor concentration peak at a known depth, and (ii) capacitance–voltage measurements for donor profiling. We cannot confirm the claims stating an extremely high permittivity of single crystalline TiO_2_. On the contrary, the permittivity of conductive, reduced single crystalline TiO_2_ is similar to that of insulating TiO_2_ established previously, with a Curie–Weiss type temperature dependence and the values in the range 160–240 along with the c-axis.

## Introduction

The static and low-frequency dielectric constant, ε, of rutile TiO_2_ has been surrounded with controversy since as early as 1952, when Nicolini^1^ reported an extremely high value of around 10,000 for the permittivity of ceramic rutile TiO_2_. Similar values of (1 − 3) × 10^4^ were later observed by Parker and Wasilik^2^ for single crystalline rutile TiO_2_. It was immediately realized, however, that such high values may be a result of an incorrectly designed experiment or an incorrect interpretation. Indeed, the method used in Ref.^2^ is based on capacitance measurements over the full thickness of the crystal, where metallic contacts are deposited on opposite sides. Parker and Wasilik^2^ suggested that, in the case of non-negligible free carrier concentration, i.e., non-negligible effective net doping, Schottky contacts can be unintentionally formed (see Supplementary Material for a description of the Schottky contact). This is particularly relevant in the case of reduced TiO_2_, where oxygen vacancies give rise to n-type doping. In such samples, the total capacitance of the crystal is determined predominantly by the depletion region associated with the Schottky contact, or “by an electron-deficient barrier layer at the electrode-crystal interface such as has been proposed to explain the action of contact rectifiers” as formulated by Parker and Wasilik^2^ with a reference to the original work by Schottky^3^. Nowadays, Schottky rectifiers or Schottky diodes, as the modern accepted term, are widely used in semiconductor science and technology.

Based on these findings, Parker^4,5^ performed new theoretical and experimental studies of ε in rutile TiO_2_ crystals. Special care was taken to prepare highly resistive TiO_2_ by “heavy oxidation” as phrased in Ref.^4^. Highly resistive, oxygen-rich rutile TiO_2_ crystals were then investigated by measuring the capacitance between a parallel plate capacitor with the crystal inside. Permittivity of 170 and 86 were measured at 300 K along with the c- and a-axes, respectively. At 1*.*6 K, the permittivity along with the c- and a-axis were deduced to be 257 and 111, respectively. No frequency dependence was observed in the frequency range 10^2^–3 × 10^6^ Hz.

Later, Samara and Peercy^6^ measured pressure and temperature dependencies of ε. Similar to the previous investigations, ε was determined from capacitance measurements. No frequency dependence was assumed, based on the findings by Parker^4^, and the measurements were performed at 100 kHz. It has been shown that data can be fitted over the whole temperature range by the modified Curie–Weiss law derived first by Barrett^7^ for perovskite-type crystals:

1 $$\varepsilon ={A}_{0}+\frac{{C}_{0}}{\frac{1}{2}{T}_{1}\mathrm{coth}\left(\frac{{T}_{1}}{2T}\right)-{T}_{0}},$$

where *T* is temperature, and *A*_0_, *C*_0_, *T*_1_ and *T*_0_ are fitting parameters. At 296 K, the permittivity along with the c- and a-axes was determined to be 166.7 and 89.8, respectively. As the temperature was decreased to 4 K, the permittivity increased to 251 along with the c-axis and to 114.9 along the a-axis. These values are close to those determined by Parker^4^.

Reports on extremely high ε of reduced single-crystalline rutile TiO_2_ continue to appear in the literature. For example, Chu^8^ has reported values in a range of 100 − 10,000. The permittivity was deduced from impedance measurements over the full crystal thickness, with gold contacts deposited on opposite sides of the sample. This concept is similar to that used in the earlier studies^2^. Recently, Li et al.^9^ reported a colossal dielectric permittivity in hydrogen-reduced rutile TiO_2_ crystals. Similar to earlier studies, ε was deduced from impedance measurements of the crystal with silver contacts. One can notice, however, that neither Chu^8^ nor Li et al.^9^ has considered formation of Schottky barriers at the metal-TiO_2_ interface and the corresponding depletion regions, which was considered by Parker and Wasilik^2^.

In this study, we intentionally form Schottky diodes on reduced single-crystalline rutile TiO_2_ and utilize their properties to deduce ε. The method involves: (i) hydrogen ion-implantation to form a donor concentration peak at a known depth, and (ii) capacitance–voltage measurements for donor profiling.

## Results and discussion

Annealing of bulk TiO_2_ in reducing and hydrogen-rich atmosphere has long been known to result in conductive, n-type bulk material (see, for instance, Ref.^10^). Two main mechanisms are believed to be responsible: (i) formation of donors assigned to oxygen vacancies (V_O_) and titanium interstitials (Ti_i_) in the reducing atmosphere^11^ and (ii) introduction of interstitial hydrogen (H_i_) donors during hydrogenation^12,13^. Besides, H_i_ can interact with acceptors and passivate them, increasing the net n-type conductivity. We have demonstrated previously^14,15^ that annealing of single-crystalline TiO_2_ wafers in N_2_ at 1100–1200 °C or in forming gas (FG), 10%at. H_2_ and 90%at. N_2_, at 600 °C leads to increase in conductivity of the wafers. In the case of heat treatment in FG, the increase can be correlated with concentration of H_i_. For annealing in N_2_, the increase in conductivity occurs without a corresponding increase in hydrogen concentration. These observations support the feasibility of the two mechanisms that involve V_O_/Ti_i_ and H_i_. The effect of hydrogen-induced donors was also used to form a pronounced donor profile at a well-defined depth by ion implantation.

In the present study, single-crystalline rutile TiO_2_ wafers with a size of 5 × 5 mm^2^ and a conductivity of < 10^−7^ Ω^−1^ cm^−1^ were heat treated in forming gas (FG) at 600 °C for 90 min or in N_2_ at 1100 °C for 60 min. As a result of the heat treatments, the conductivity of the TiO_2_ samples has increased to 0.01–0.07 Ω^−1^ cm^−1^. Subsequently, Pd contacts with a diameter of around 400 μm were deposited. A stack of Ti/Al layers was deposited over the whole back side surface of the samples as the back side contact. Current–voltage (I–V) measurements reveal a rectification of up to eight orders of magnitude, indicating that Pd Schottky diodes are formed. The I–V measurements show that *at the reverse bias conditions* the dc impedance of the Pd Schottky contact dominates over the bulk dc impedance of the samples. It should also be emphasized that the size of the Pd contacts is significantly less than the sample size. Further measurements are performed at the reverse bias, when the impedance associated with the Schottky barrier and the depletion region is dominant.

**Figure 1:**
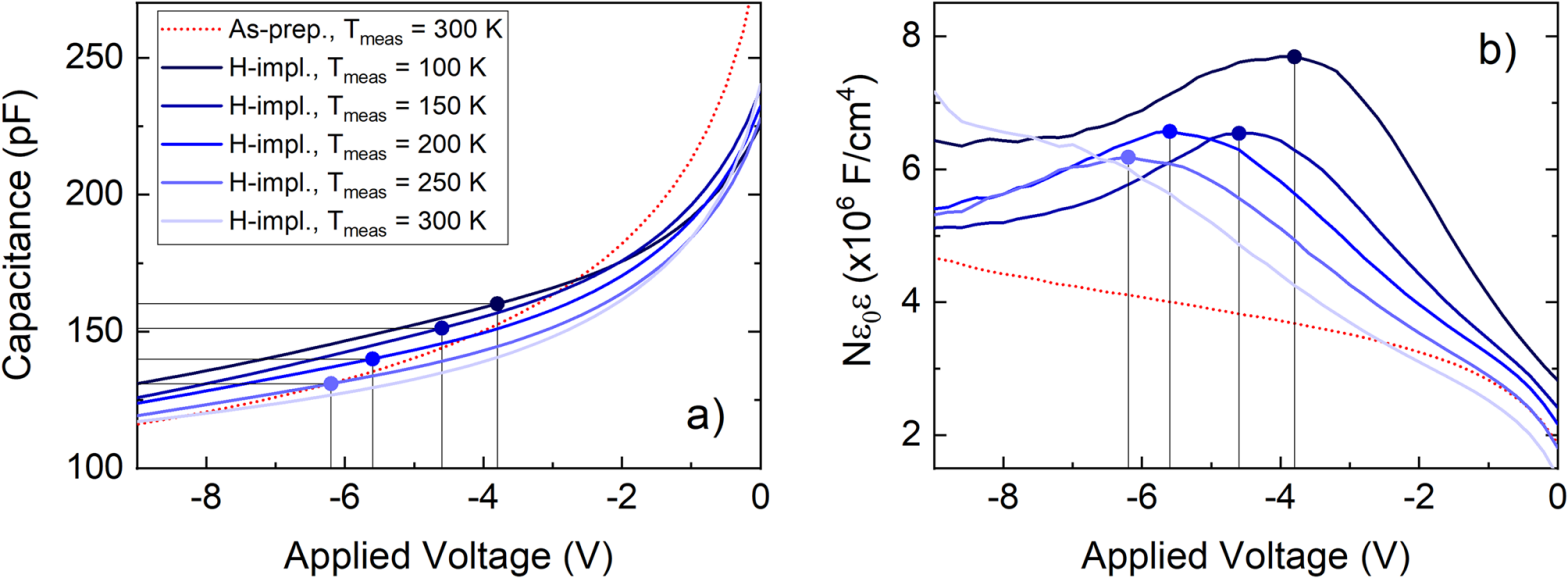
Capacitance **(a)** of a TiO_2_-N_2_ sample (probing frequency 60 kHz) and the *N*(*V*)ε_0_ε product **(b)** as functions of applied voltage. The red, dotted curve shows the as-prepared sample, i.e., after the N_2_ heat treatment but prior to H^+^ implantation. The blue, solid curves are for the H-implanted sample measured at different temperatures (*T*_meas_). The peaks in *N*(*V*)ε_0_ε are indicated by the drop-down lines. The corresponding capacitance values, obtained at the same applied voltages, are also marked in **(a)**.

Capacitance–voltage (CV) measurements are a well-established technique for probing the depth distribution of donors and acceptors. Figure 1 depicts CV measurements at different temperatures (*T*_meas_) for a sample annealed in N_2_ at 1100 °C for 60 min (TiO_2_–N_2_). The CV measurement performed prior to H^+^ implantation is shown with red, dotted curve (Fig. 1a). The measurement is carried out at 300 K and shows decreasing capacitance with increasing reverse bias, in accordance with the expected dependence for a Schottky diode (see Supplementary Material). Blue, solid curves represent measurements recorded after the sample was implanted with 200-keV H^+^ to a dose of 3 × 10^13^ cm^−2^. The different blue curves represent measurements recorded at different *T*_meas_. Figure 1a shows that capacitance of the Schottky diode decreases with increasing reverse voltage, *V*, as expected for a diode.

Within the depletion approximation (see Ref.^16^ or Supplementary Material), one can derive the following expression:

2 $$N(V){\upvarepsilon }_{0}\upvarepsilon =\frac{{C}^{3}}{q{A}^{2}}{\left(\frac{dC}{dV}\right)}^{-1},$$

where *N*(*V*) is the doping concentration at the depth of the depletion region for a given *V*, ε_0_ is the vacuum permittivity, ε is the relative permittivity, *C* is the capacitance at the given *V*, *q* is the electron charge and *A* is the area of the diode. Since ε_0_ and ε do not depend on *V*, the product *N****(****V*)ε_0_ε will maintain the shape of *N*(*V*).

Figure 1b displays *N*(*V*)ε_0_ε as a function of *V*. For the as-prepared sample prior to H^+^ implantation, the data reveal a somewhat non-uniform, but monotonous *N*(*V*)ε_0_ε as a function of *V*. Hydrogen implantation leads to formation of a pronounced peak in *N*(*V*)ε_0_ε. One can thus identify the voltages at which the edge of the depletion region reaches the peak of *N*(*V*)ε_0_ε, as indicated in Fig. 1b with filled circles. For example:

- For *T*_meas_ = 100 K, the depletion region edge reaches the donor concentration peak at *V*_peak_=−3*.*8 V (Fig. 1b). This voltage corresponds to the capacitance *C*_peak_= 160 pF, indicated in Fig. 1a.
- For *T*_meas_ = 250 K, the depletion region edge reaches the donor concentration peak at *V*_peak_=−6*.*2 V (Fig. 1b). This voltage corresponds to the capacitance *C*_peak_= 131 pF, indicated in Fig. 1a.

On the other hand, the depth of the implantation peak for hydrogen-induced donors is known from secondary ion mass spectrometry (SIMS) measurements: *d*_peak_ = 0*.*97 µm (see Supplementary Material).

Within the depletion approximation, the capacitance, *C*, and the depletion depth, *d*, are related as *C* = *ε*_0_*εA/d*. One can thus find ε from:

3 $$\upvarepsilon =\frac{{C}_{\mathrm{peak}}{d}_{\mathrm{peak}}}{{\upvarepsilon }_{0}A}.$$

The results of the analysis from Fig. 1 and Eq. (3) are summarized in Table 1. No frequency dependence has been observed within the range between 1 kHz and 1 MHz (see Supplementary Material).

**Table 1:** Data used for analysis of temperature dependence of ε for the TiO_2_-N_2_ sample with CV data plotted in Fig. 1. The implantation depth *d*_peak_ = 0*.*97 µm. The diode area *A* = 8.3 × 10^–4^ cm^2^.

Temperature, K	*V*_peak_, V	*C*_peak_, pF	ε
100	− 3*.*8	160	211
150	− 4*.*6	151	199
200	− 5*.*6	140	185
250	− 6*.*2	131	173

It should be noted, however, that in some cases we could not perform this analysis at the given experimental conditions. The method relies on the implanted hydrogen profile being well within the depletion region of the Schottky diode. This requires a careful choice of the implantation energy, in order to provide a suitable implantation depth. However, the depletion region depends on (i) the concentration of ionized donors, (ii) the Fermi level in the material, and (iii) the permittivity. Since all these factors can change with temperature, a given implantation depth may not be suitable for all temperatures. In addition, in order to reliably resolve the implanted peak, the concentration of implanted hydrogen donors should significantly exceed the background donor concentration. At the same time, the concentration of the implanted hydrogen cannot be too high, in order to avoid a strong effect on the depletion region depth and/or to prevent phase transformations. One of the examples is illustrated in Fig. 1b, where the curve for *T*_meas_ = 300 K does not reveal a well-defined donor concentration peak. In this particular case, two effects are believed to occur: (i) the concentration of the background ionized donors appears to be comparable to the concentration of the implanted donors, and (ii) the implanted donor peak is too close to the maximum depletion depth. However, at lower temperatures, *T*_meas_ ≤ 250 K, the apparent concentration of ionized background donors decreases, and the implantation peak becomes more prominent.

**Figure 2:**
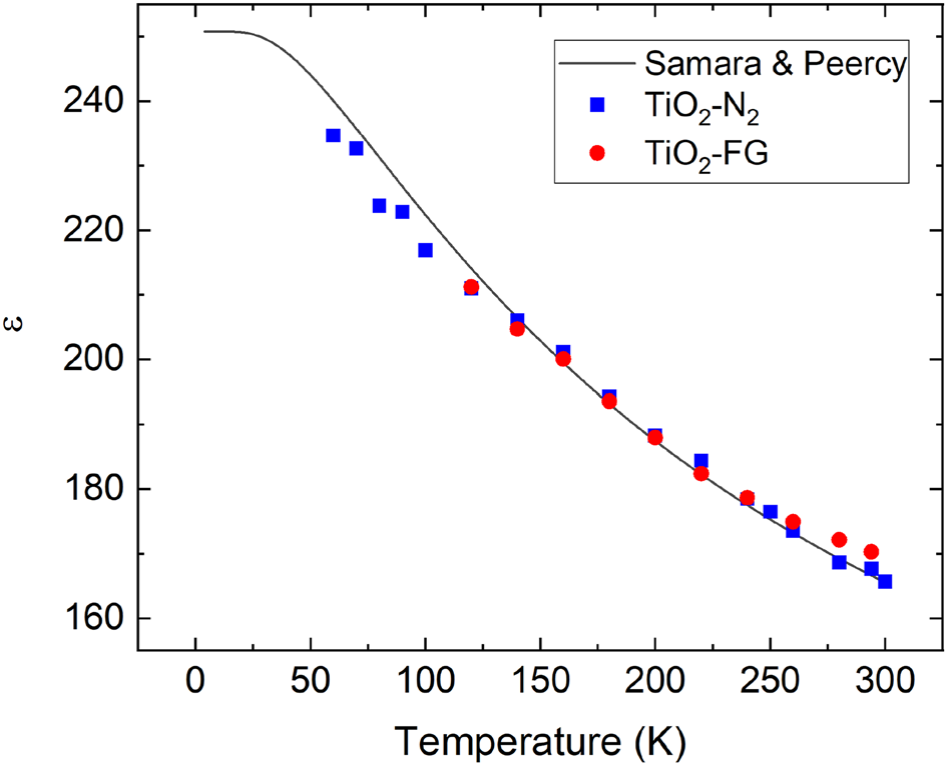
Temperature dependence of the c-axis permittivity (*ε*) of conductive, n-type TiO_2_ obtained after heat treatments in FG and N_2_; and a modified Curie–Weiss dependence with the parameters determined by Samara and Peercy^6^ (solid curve).

The analysis described by Fig. 1 and Eqs. (2) and (3) has been applied for more detailed studies of ε in a number of samples annealed in FG (TiO_2_-FG) and the TiO_2_–N_2_ samples. Figure 2 demonstrates that ε determined in the present study is very close to that determined for insulating, oxygen-rich rutile TiO_2_ by Samara and Peercy^6^. For reduced TiO_2_, ε along with the c-axis is in the range 160 − 240 for temperatures 50–300 K. It decreases as temperature increases and can be described by the modified Curie–Weiss law. We do not observe a significant difference in ε between reduced TiO_2_ (annealed in N_2_ at 1100 °C) and reduced-hydrogenated TiO_2_ (annealed in FG at 600 °C).

## Conclusion

In conclusion, measurements of the permittivity, ε, in conductive single-crystalline TiO_2_ are challenging and causing controversy. We propose a method for deducing ε from capacitance measurements. The method involves formation of Schottky barrier diodes and hydrogen implantation. The implantation results in a well-pronounced donor concentration profile, corresponding to the implanted hydrogen profile. The donor profile is then characterized using capacitance–voltage measurements, and ε can be deduced. We observe that ε of reduced, conductive rutile TiO_2_ is similar to that of oxygen-rich, insulating rutile TiO_2_ established previously. We cannot confirm claims of colossal dielectric permittivity in hydrogenated and reduced rutile single-crystalline TiO_2_.

The Research Council of Norway is acknowledged for the support to the Norwegian Micro- and Nano-Fabrication Facility, NorFab, project number 295864. Financial support by the Research Council of Norway via the EEA-JRPRO-NO-2013-1 European Project (PERPHECT), the Research Center for Sustainable Solar Cell Technology (FME SUSOLTECH, project number 257639), and the Norwegian PhD Network on Nanotechnology for Microsystems (project number 221860/F60), is gratefully acknowledged. Financial support by the Faculty of Mathematics and Natural Sciences at the University of Oslo via the strategic research initiative FOXHOUND is gratefully acknowledged.

## Methods

### A. Samples

The study was performed on Verneuil-grown rutile TiO_2_ single crystals with a surface orientation of (001), purchased from *Shinkosha Co., Ltd.*^17^. As-received crystals were 0*.*5 mm thick with double-side polished surfaces, nominally undoped, transparent, and semi-insulating with a conductivity of *σ* < 10^−7^ Ω^−1^ cm^−1^. Conductive *n*-type TiO_2_ samples of bluish colour were obtained by heat treatments in forming gas (FG) flow (N_2_ + H_2_ with [H_2_]*/*[N_2_] ≈ 1/9) at 600 °C for 90 min (hydrogenating and reducing heat treatment) or in N_2_ flow at 1100 °C for 60 min (reducing heat treatment). The heat treatments resulted in increased conductivity of the samples in the range 0.01–0.07 Ω^−1^ cm^−1^. Subsequently, circular 150-nm thick Pd contacts with a diameter of around 400 μm were deposited through a shadow mask. A back side contact was made by depositing a stack of Ti/Al layers over the whole back side surface of the wafer: (1) a 10-nm Ti-layer on TiO_2_ and (2) a 150-nm Al-layer on Ti. This resulted in Schottky diodes with a rectification of up to eight orders of magnitude^18^. After initial electrical measurements, the samples were implanted at room temperature with 200-keV H^+^ ions to different doses in the range 6 × 10^12^—3 × 10^14^ cm^−2^.

### B. Experimental set-up

Secondary ion mass spectrometry (SIMS) measurements were performed using a Cameca IMS 7f spectrometer with a primary beam of 15-keV Cs^+^ ions. A constant erosion rate was assumed for depth-calibration, where the crater depths were measured using a DekTak Stylus Profilometer.

After the electrical measurements, each Schottky diode was measured by the profilometer for accurate determination of the diode area.

Capacitance–Voltage (CV) measurements were carried out under dark conditions at temperatures in the range between 20 and 300 K using an Agilent 4284A LCR Meter at six different probing frequencies between 1 kHz and 1 MHz and with a probing AC amplitude of 30 mV. The LCR-meter was used in two modes: (1) so-called parallel mode (*C*_p_–*G*_p_) and (2) so-called series mode (*C*_s_–*R*_s_).

## Supplementary Information

Supplementary Information.

## References

[1] Nicolini L (1952). A new dielectric material. Nature.

[2] Parker RA, Wasilik JH (1960). Dielectric constant and dielectric loss of TiO_2_ (rutile) at low frequencies. Phys. Rev..

[3] Schottky W (1942). Vereinfachte und erweiterte Theorie der Randschicht-gleichrichter. Z. Physik.

[4] Parker RA (1961). Static dielectric constant of rutile (TiO2), 1.6-1060 K. Phys. Rev..

[5] Parker RA (1961). Lorentz corrections in rutile. Phys. Rev..

[6] Samara G, Peercy P (1973). Pressure and temperature dependences of the Raman-active phonons in SnO_2_. Phys. Rev. B.

[7] Barrett JH (1952). Dielectric constant in perovskite type crystals. Phys. Rev..

[8] Chu C (1970). New ordered phases of slightly reduced rutile and their sharp dielectric absorptions at low temperature. Phys. Rev. B.

[9] Li J, Li F, Zhu X, Lin D, Li Q, Liu W, Xu Z (2017). Colossal dielectric permittivity in hydrogen-reduced rutile TiO2 crystals. J. Alloys Compds..

[10] Becker JH, Hosler WR (1965). Multiple-band conduction in n-type rutile (TiO_2_). Phys. Rev..

[11] Deák P, Aradi B, Frauenheim T (2015). Oxygen deficiency in TiO2: Similarities and differences between the Ti self-interstitial and the O vacancy in bulk rutile and anatase. Phys. Rev. B.

[12] Brant AT, Yang S, Giles NC, Halliburton LE (2011). Hydrogen donors and Ti3+ ions in reduced TiO2 crystals. J. Appl. Phys..

[13] Herklotz F, Lavrov EV, Weber J (2011). Infrared absorption of the hydrogen donor in rutile TiO2. Phys. Rev. B.

[14] Zimmermann C, Bonkerud J, Herklotz F, Sky TN, Hupfer A, Monakhov E, Svensson BG, Vines L (2018). Influence of annealing atmosphere on formation of electrically-active defects in rutile TiO2. J. Appl. Phys..

[15] Weiser PM, Zimmermann C, Bonkerud J, Vines L, Monakhov EV (2020). Donors and polaronic absorption in rutile TiO2 single crystals. J. Appl. Phys..

[16] Blood, P., Orton, J. W. *The Electrical Characterization of Semiconductors: Majority Carriers and Electron States* (Academic Press, 1992).

[17] *Verneuil-Grown r-TiO*_*2*_* from Shinkosha*. https://www.shinkosha.com/english/sehin/2_03.html. Accessed 05 Dec 2019.

[18] Bonkerud J, Zimmermann C, Herklotz F, Weiser PM, Aarholt T, Verhoeven EF, Vines L, Monakhov EV (2020). Fabrication and characterization of Schottky barrier diodes on rutile TiO2. Mater. Res. Exp..

