# Supplementary material for: On the permittivity of titanium dioxide
Source: Sci Rep. 2021 Jun 14;11:12443. doi: 10.1038/s41598-021-92021-5 (PMC8203734; doi:10.1038/s41598-021-92021-5)
Supplement: Supplementary file 1 — Supplementary Information. [file 41598_2021_92021_MOESM1_ESM.pdf]

Supplementary Material for:

On the permittivity of titanium dioxide

Julie Bonkerud, Christian Zimmermann, Philip Michael Weiser, Lasse Vines, and Eduard V. Monakhov

I. Schottky contacts and depletion approximation

When a metal is brought into contact with a semiconductor, a voltage can build up at the interface. The value of this built-in voltage is defined by the difference in the Fermi levels between the metal and the semiconductor. Due to the built-in voltage, a barrier layer is formed, where the carriers are driven out, and the layer becomes depleted of carriers. This layer is normally called a depletion layer. The contact, where the difference in the Fermi levels between the metal and semiconductor is significant and the depletion region is formed, is normally called a Schottky contact. Formation of a Schottky contact and the depletion region is described, for example, by Kittel [1].

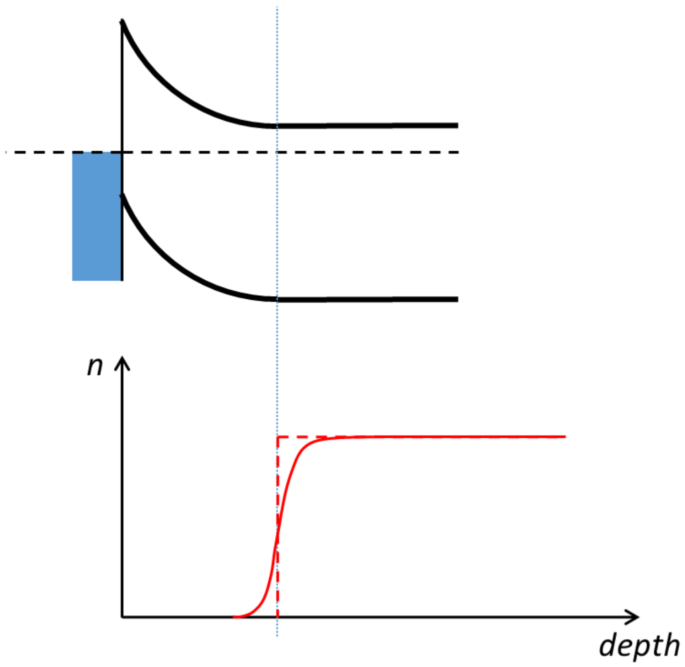

Fig. S1. Band structure of the Schottky contact (top) and the carrier distribution (electrons in this example) over depth (bottom). The realistic distribution is shown with the solid curve, the distribution within the depletion approximation is depicted with the dashed curve.

Depletion approximation

Generally speaking the edge of the depletion region is poorly defined since the carrier concentration changes gradually, determined by a combination of the band bending and Fermi-Dirac statistics. The *depletion approximation* assumes that (i) the carrier concentration changes abruptly at the edge of the depletion region, e.i., one can accurately define the depletion region, and (ii) the carrier concentration within the depletion region is zero. Fig. S1 illustrates the depletion approximation for a Schottky contact. This approximation is used in all books on semiconductor devices, and has proven to be very precise [2,3,4].

For a uniform doping concentration, the depletion approximation results in the well-known expression for the depletion region depth, *d*:

$$d = \sqrt{\frac{2\varepsilon_0\varepsilon}{eN}(V_{bi} - V)}\,,$$

where ε_0 is the permittivity of vacuum, ε is the relative permittivity, *e* is the electron charge, *N* is the effective doping concentration, V_{bi} is the built-in voltage, *V* is the external applied voltage. *V* is positive for forward bias and negative for reverse bias. The capacitance, *C*, of the Schottky contact/diode is then defined by the depletion region width:

$$C = \frac{\varepsilon_0\varepsilon A}{d}\,,$$

where *A* is the area of the Schottky contact.

In the case of nonuniform doping concentration, one can derive the following dependence [2,3,4]:

$$N(V)\varepsilon_0\varepsilon = \frac{C^3}{qA^2}\left(\frac{dC}{dV}\right)^{-1},$$

where $N(V)$ is the effective doping concentration at the edge of the depletion region for the given *V*. This formula is used in the study.

II. Hydrogen implantation and hydrogen concentration profile

Heat treatments were performed in forming gas (FG) flow (N_2 + H_2 with $[H_2]/[N_2] \approx 1/9$) at 600˚C for 90 min, labeled as TiO_2-FG, or in N_2 flow at 1100˚C for 60 min, labeled as TiO_2-N_2. After the heat treatments and deposition of 150-nm thick Pd contacts, the samples were

implanted at room temperature with 200-keV H^+ ions to different doses in the range 6×10^{12} - 3×10^{14} cm^{-2}. The implantation depth in TiO_2 was predicted to be 0.93 μm by Stopping and Range of Ions in Matter (SRIM) simulations [5].

Hydrogen distribution was then measured by secondary ion mass spectrometry (SIMS). Since SIMS is a destructive method, only one sample (TiO_2-FG) was selected. The measurements show a significant presence of hydrogen (Fig. S2) due to the treatment in FG and implantation. It should be noted, however, that hydrogen in TiO_2 can exist in different atomic configuration, forming complexes with other defects. Thus the total hydrogen concentration, determined by SIMS, may not be equal to the net donor concentration. The implantation results in a pronounced peak in the hydrogen concentration at d_{peak}=0.97 μm, as can be seen from the SIMS data in Fig. S2. This implantation depth is within 4% accuracy of the depth predicted by SRIM (0.93 μm).

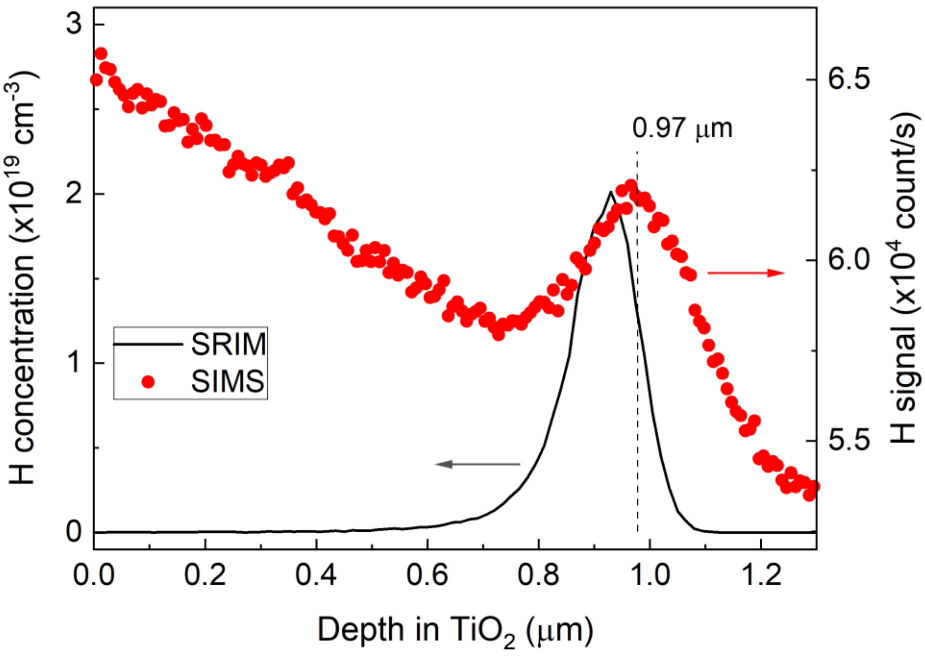

Fig. S2. Hydrogen distribution predicted by SRIM simulations for 200-keV H^+ implantation with a dose of 3×10^{14} cm^{-2} and the hydrogen signal (counts/s) in SIMS measurements as a function of depth in TiO_2.

III. Frequency dependence of the dielectric constant

Frequency dependence of ε was investigated in the range 1 kHz−1 MHz. The measurements were performed in two modes: (1) so-called parallel mode (C_p-G_p) and (2) so-called series mode (C_s-R_s), where the series resistance is significant.

Fig. S3 shows the frequency dependence of capacitance-voltage (CV) measurements recorded at T_{meas} = 150 K for the TiO_2-N_2 sample after H^+ implantation. T_{meas} = 150 K is chosen as being about the middle of the studied temperature range (20 – 300 K), and it is representative for other temperatures. Measurements in the C_p−G_p mode do not reveal any dispersion for frequencies in the range 1–60 kHz (Fig. S3a). The product $N\varepsilon_0\varepsilon$ exhibits a maximum at the same voltage (−5.0 V) for all the frequencies (Fig. S3b). An indication of dispersion can be observed for a frequency of 0.25 MHz, and a strong dispersion is revealed for 1 MHz (Fig. S3a). We, however, attribute this dispersion to a measurement artifact due to limitations of the C_p−G_p model, rather than frequency dependence of ε.

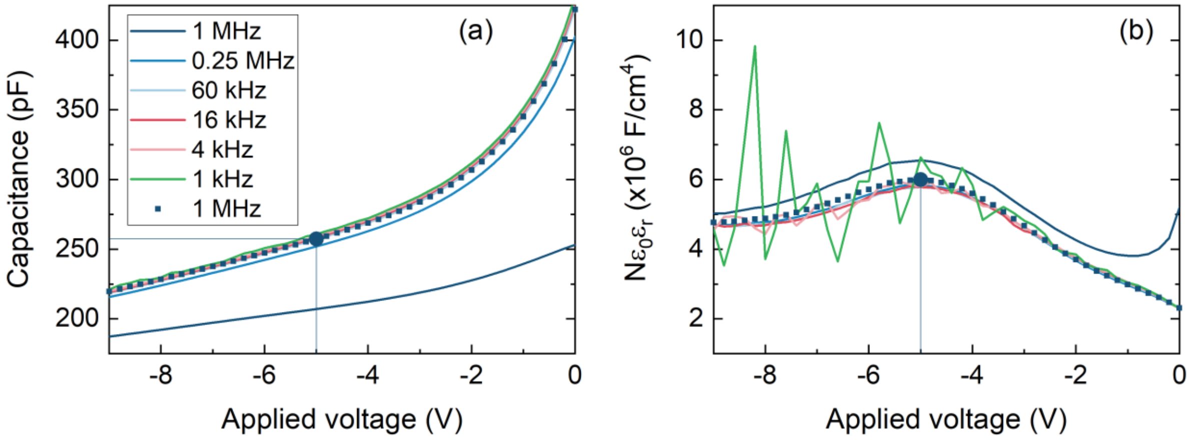

Fig. S3. Capacitance (a) and the product $N\varepsilon_0\varepsilon$ (b) for different frequencies as functions of voltage for a hydrogen-implanted TiO_2-N_2 sample. The solid curves are data from measurements recorded assuming a $C_p - G_p$ circuit, while squares represent data measured assuming a $C_s - R_s$ circuit. The voltage, V_{peak}, corresponding to the peak in $N\varepsilon_0\varepsilon$ is indicated by a drop-down line in (b). The corresponding value of capacitance, obtained for the given V_{peak}, is also marked in (a).

The impedance of the depletion region depends on the probing frequency, f, as $(2\pi fC)^{-1}$. With increasing f, the impedance of the depletion region decreases and can become comparable to or less than the serial resistance of the sample. In this case, the C_p−G_p model is no longer applicable, and the C_s-R_s model should become valid. The measurements in the C_s−R_s mode at 1 MHz (symbols in Fig. S3a) indeed remove the frequency dispersion. We conclude, thus, that ε has no frequency dependence in the range 1 kHz – 1 MHz, and the results reported for f = 60 kHz in the main text are valid for all frequencies.

References

[1] C. Kittel, "Introduction to Solid State Physics", Chapter 17, p. 506, 8th ed., Wiley (2005).
[2] P. Blood and J. W. Orton, "The Electrical Characterization of Semiconductors: Majority Carriers and Electron States", Academic Press (1992).
[3] S. M. Sze and Kwok K. Ng, "Physics of Semiconductor Devices", Wiley (2007).
[4] D. K. Schroder, "Semiconductor material and device characterization", Wiley (2006).
[5] J. F. Ziegler, M. D. Ziegler, and J. P. Biersack, Nuclear Instruments and Methods in Physics Research B **268**, 1818 (2010).
